# Juvenile Delinquency and Degeneration in the Upper Classes of Society

**Published:** 1849-07-01

**Authors:** W. M. Bush

**Affiliations:** Medical Superintendent of Sandywell Park, near Cheltenham


### JUVENILE DELINQUENCY AND DEGENERATION IN THE UPPER CLASSES OF SOCIETY.

BY W. M. BUSH, M.D., F.L.S.,

*Medical Superintendent of Sandywell Park, near Cheltenham.*

THE increased attention bestowed within the last fifteen years upon the nature and causes of insanity, has resulted in other benefits than an improved treatment of the insane. It has converted the crude, empirical notions which previously existed, and enveloped that disease in mystery, into a science, shedding light upon the path of the inquirer, and revealing truths which had been long concealed from the world.

Among the many benefits likely to accrue to society from the more enlightened views of medical men on this subject, is the success which has attended their investigation into the operation of various agencies upon the human mind; whereby we, becoming better acquainted with those circumstances which favour or disturb the healthy action of the brain, may learn to conduct ourselves in conformity with those rational principles which such knowledge necessarily provides.

The more we inquire into the influence of disturbing agencies on the mind, the more convinced must we be of the danger of any protracted exposure to them, and the necessity of avoiding, at any sacrifice, the numerous ills which follow in their train. It is in pointing out these disturbances, and their effects upon the mind and general health, that the improved modern notions of insanity so much excel; and it is in accordance with these notions that attention is directed to the important social question which forms the subject matter of the following essay.

If, then, the metaphysical truths revealed by medical psychology, or the science of mind in relation to health, be of use as a guide to the general preservation of the mind, the recognition of those truths certainly belongs to early mental cultivation, and more especially so to the management of weak intellect, or defective morals developing themselves in early life.

Improved pathological science confirms this opinion, and explaining as it does the cause of those mental and moral peculiarities observable in many children, forcibly points out the mischievous and dangerous consequences of subjecting such as are of a peculiar nervous conformation to the ordinary curriculum of a school.

It is a most certain though melancholy fact, in confirmation of the above remark, that there are many children brought prematurely to a miserable death, or to a state of protracted idiocy, through the ill-directed zeal of their instructors, who being ignorant of the principles of mental pathology—mistaking incapacity for idleness, precocity for vigour, and perversion for immorality—increase the very mischief they profess to avert.

How important, then, in such cases, becomes an intimate and practical acquaintance with the phenomena arising out of the connexion of mind and matter!—with their several reactions, healthy and diseased. For the fate of every young person who is considered a "genius," or "dull," or "wayward," will be involved in the management to which he is subjected in tender life. It will depend on this whether he shall be a misery to himself and a disgrace to others, be prematurely cut off by mental and moral decay, and even by physical death, or (under an appropriate discipline, founded on the acknowledged principles of medical psychology) shall enjoy the possession of those moral attributes which adorn the individual, and make him an useful member of society.

To explain the subject more fully, it may be useful briefly to sketch the ordinary career of one boy at least in almost every school. Incapable, from *defective organization*, of acquiring that amount of knowledge which his tutor imperatively, and often dogmatically, demands, his parents expect, and his age would warrant, he is condemned as "idle," and punishment is used to excite physically, what is morally impossible—viz., certain mental manifestations from a brain too weak or disordered to evince them. From that moment his troubles and his ruin may be dated: his slight remaining energy (always fugitive) is destroyed by frequently-repeated punishment; dismayed and listless, he becomes the opprobrium of the school and the jest of his companions—a mingled feeling of injury and shame degrades him in his own estimation, and he is at length *made*, what he is but too truly called, "incorrigible"—neglected and discouraged, the slender fabric of morality breaks down, and, as though it were designed to complete and perpetuate his ruin, the last stone of hope is removed, every avenue of reformation closed, by expulsion from the school to a home where he is unwelcome, for the commission of some vicious act which he has lost the mental power to resist, or the moral sense to appreciate.

In such a career, we see the gradual destruction of a fragile vessel, hastened by the ambition of the world, till it comes broken to the asylum or the grave—we trace the progressive decline of an immortal creature to usages founded in error, and maintained by an almost monastic bigotry—we observe each human attribute, though weak, still noble, crushed by conventional exactions too onerous to be borne; till at length, the course being run, the school ordeal passed, we find the victim of all these destructive influences (bereft of intellect and sapped in morals) left to dwell in the mental wilderness which an over-zealous system has produced.

It may be urged that an extreme case has been selected for the exposure of this evil, that the consequences arising from over-pressure of the mind do not accumulate within so brief a space of life, nor arise and terminate in the rapid course described, and that the dulness and perverseness of youth have been overcome by the severity now condemned. But these are deceptive arguments, the mere excuses of ignorance, the temporary evasions of a truth which will only peal the louder on the ear of conviction the longer it is postponed. The terrible consequences may not, indeed, be fulfilled at so early an age; for as jealous nature will, when violated, attempt her own defence, it is found that the imbecile mind when young will often put forth some buds of promise, affording to the too sanguine parent hopes of future blossom. A few dim rays of reason will now and then break the uniform obscurity of the dullest mind, and may be hailed by some as the forerunner of a more constant mental sunshine.

But let us not be deceived by such flattering appearances. Let us remember that there are degrees of imbecility—that with only a little less weakness of mind, and a little more constitutional vigour, the consequences predicted will not reveal themselves at so young an age, but at a later period; not perhaps at school, but at the university—in the counting-house—in the tavern—in haunts of vice—wherever can be traced the steps of mental prostitution or moral depravity.

It may not be very agreeable for many parents and instructors to be informed of what daily experience proves to the medical practitioner—viz., that with the exception of congenital idiots, the great majority of idle and profligate young men have been made what they are by mismanagement at school—by the *indiscriminate* system to which boys of every temperament and shade of mind are subjected; that so long as our present system of education is adapted to only one set of individuals—to those only having a sound mind in a sound body—and that so long as the phenomena of those complicated and ever-changing reactions between mind and body continue unrecognised in the management of youths, our schools will contribute, as they now do, to break the charm that sanctifies the English home, and to destroy the beneficial influence which rank and fortune ought always to exercise upon the community.

If there be any truth at all in what has been stated, it becomes incumbent on the part of every parent to test it by his own experience, and to inquire whether the perverseness of his child does not date its origin from the causes assigned?—whether the popular remedy of the school has proved the panacea he expected?—and if not, whether some other system, more rational and certain, cannot be substituted? Before proceeding to answer these questions and to propose a more satisfactory plan of management, it will be necessary to describe, in more definite language, those distinctive characteristics which mark the individual cases unsuited to modern scholastic discipline. As it is at the age of ten years, or nearly so, that any marked peculiarities in mind or body begin to show themselves, and as it is at this time also that they require so much attention, this age and the following six years will be selected as the period to which these remarks more particularly apply.

Although there are many morbid conditions of the body which incapacitate youth for the ordeal of schools and the exactions of modern education, the present article will be confined to a notice of those only which exist in the *nervous system.*

The subject will be considered in reference to the existence of general want of tone, with excessive or diminished nervous energy.

### 1*st. Excessive irritability of the nervous system, with general want of mental and bodily vigour.*

1. Idiopathic epilepsy, or convulsive fits with unconsciousness, arising from constitutional causes, being the great type of all convulsive affections, is placed at the head of the list. Childhood, from infancy upwards, is liable to every form of convulsions. The same diathesis, the same nervous condition which produces a momentary stiffening of the limbs, a transient unconsciousness, or a single sudden scream, may be aggravated by undue excitement of the great nervous centres to such a degree as to excite the dreadful struggles of epilepsy. A few only, however, will be selected from the great variety of fits to which boys at the above age are more frequently liable.

2. Many boys at the age of eight or ten years will be found to have contracted various unsightly tricks of a purely nervous character, such as blinking, stammering, a spasmodic action of the muscles of the face, and an awkward movement of the limbs. It will be found that all these eccentric muscular movements are increased by moral emotions; for springing as they do from an oversensitive condition of the nervous system, whatever excites the great centres of that system (the brain and spinal cord) will be sure to aggravate the symptoms. This morbid sensibility of the nerves is exhibited in mental as well as bodily peculiarities, and we accordingly find these subjects to present a character different to, or in the extreme of, what is observed in other boys. They are reserved or fond of solitude; do not enter at all into juvenile sports, or else engage in them with excessive ardour. They are easily cowed, irascible, timid to an excess, terrified at darkness, and are either unusually dull, or show a precocity of intellect which very speedily decays. It would seem as though nature were in a state of morbid intensity in these cases; as though the puerile character were exaggerated; for whereas all boys are by nature awkward and rude in their manners, these are more so, they are eccentric; and where others are shy, these are confused; or where timid only, these are altogether overcome by terror. It is in these cases, too, that the *imitative character* common to all children, becomes so prominent as to amount to disease; for it not only keeps up those eccentric habits incidental to innate nervousness, but leads to the adoption of such as are observed in others; and moreover, extending its influence to the weakened moral faculties, induces them to follow the more easy course of a bad than a good example.

3. A very remarkable and—because so seldom regarded by parents or instructors—a very dangerous fit is mentioned by Dr. J. Conolly, of Hanwell, who describes it as follows:—"Sleepwalking and short attacks of a cataleptic kind occurring in young persons, are, I fear, often precursors of complete epilepsy, which supervenes after the age of puberty. In the cataleptic seizures to which I allude, the consciousness of the little patient is lost for a few seconds, or for nearly a minute, the eyes become fixed, and the head is moved gently up and down, but the patient does not struggle or fall. Reading, or any other occupation, is of course interrupted, but soon, after a little appearance of confusion, resumed. The conjunction of such affections with marked mental peculiarity in young persons is, consequently, alarming, foreboding epilepsy, uncertain life, or derangement of mind."

In this case, described by Dr. Conolly, of the early indications of approaching epilepsy, we see an example of the same nervous conformation, only a little more developed, as that mentioned in our second case, and which may be designated the convulsive temperament. In that case, (No. 2,) merely a constant and uniform eccentricity of muscular movement, accompanied with certain moral and intellectual peculiarities, without any absolute fit or loss of consciousness, and it is the presence of these two latter symptoms which shows a greater intensity only of the same pathological condition.

4. But there is still another form of this morbid state of the nervous system, in which likewise neither voluntary motion nor consciousness are prominently implicated, but in which the convulsive temperament is manifested chiefly in the moral emotions. We see that youths who, without any particular sign of disturbance in their ideas or consciousness, and without any struggle or muscular action beyond what is consistent with the activity of the motion excited, will be seized with a sudden and unaccountable paroxysm of passion under the most trifling circumstances, in which they will throw knives or other dangerous weapons at the party that evoked the paroxysm—will be impelled by this over-excited energy to do acts of the most reckless daring—will jump headlong into the deepest water, although they cannot swim—will suspend themselves, in simple bravado, from a parapet or window-sill with a perilous depth beneath—will risk and lose their lives in the wildest experiments with gunpowder and firearms—will take delight in torturing dumb animals, or even their playmates—and will, under the influence of this ecstatic impulse, even hang themselves in wanton play. It is in youths of this excitable temperament that we find that remarkable propensity to tell untruths, not for any purposes of concealment, but merely for the sake of exaggerating their own delinquencies. They will boast of having achieved things physically impossible, of having property and relations so preposterously grand, that were the mind really unsound they would be delusions. But they are not the delusions of an insane mind, for they do not believe the statements of which they are themselves the authors; nor can they be regarded as ordinary lies, as they are, for the most part, motiveless.

Now, all this irregular action may be considered as "perverted energy"—a kind of moral epilepsy—as an instance of the convulsive temperament, exhibiting itself in that part of the nervous system which presides over the animal energies and moral feelings.

Bearing in mind that the same morbid condition of the nervous system prevails in every form of convulsive disease, that the varieties of these diseases are only modifications of the intensity of that condition, and that the differences of symptoms are referable to the portions of that system most energetically attacked, we have a simple explanation of all the phenomena attending them. Ordinary epilepsy is the most intense form of this class of diseases, for in it the unconsciousness of mind, the convulsion of the muscles, and the suspension of the will—the grand features of all these nervous affections—are the most complete.

In that modification of the disease, just alluded to under the name of moral epilepsy, the morbid condition, though identical, is far less severe, for in it we find no muscular convulsion, but only acts of recklessness; we detect no greater suspension of the faculties, no more unconsciousness than amounts to an insensibility to danger; and so we proceed down to the most primitive and simple form of nervousness, tracing in all of them modifications of the same cause and symptoms. It is very probable that the same pathological condition of the nervous system exists in what has been called "moral epilepsy," as in "moral insanity" modified by age, and the circumstances concomitant with it.

Whenever those peculiar phenomena, of which the nervous system appears to be the source, whether they be electrical, or the vital principle itself, or some ethereal essence—whenever those vital manifestations, which all physiologists attribute to the nervous system, under the name of "vis nervosa," accumulate in greater quantity than is required for the use of the individual, they exhibit themselves in diseased action; in other words, Nature sets to work to dispose of their superfluity as useless and detrimental.

It may be here remarked, that this excessive activity is no more an evidence of tone in the nervous system than it is in the vascular, for we may with as much justice argue, that the quickened pulse in fever is an indication of increased vital power, as to say that excessive nervous energy should be attended with corresponding *power* in the character of the individual. It is that occult *cause* which supplies more nervous stimulus than is required—it is this which constitutes the disease, and we see, therefore, that activity or intensity is no test of power. The familiar instance of precocious intellect observable in some children, which springing up quickly from a weak root, soon shows its origin in its speedy decay, may be adduced in support of this opinion.

We have, then, it would seem, some clue to the origin of all convulsive affections; but we are for the most part ignorant why any particular medium should be selected for the escape of this superfluity. In a few instances, we may discover some explanation for the choice, as in the case before us, where it would appear that that portion of the nervous system most completely developed, is selected as the readiest and most appropriate channel for the escape of superfluous energy. In youths, those portions of the nervous system which preside over the animal and moral feelings are more developed and more active than the intellectual, and it is accordingly through such channels that any morbid exuberance of nervous energy displays itself, as in "moral epilepsy;" but in the adult, where the intellectual development prevails, we find the intellect (partially) as well as the morals implicated, as in "moral insanity." The judgment (discretion) must be impaired to constitute moral insanity; but in youth, there being little or no judgment to impair, we ascribe its wild impulses to an epileptic condition of that part of the nervous system which presides over the animal emotions. That the preceding explanation has some foundation in nature, we have only to refer to a disease characterized by an inordinate amount of blood in the vessels, called "phlogosis," in which we see Nature attempt a similar proceeding for getting rid of a superfluity of blood, by setting up that tumultuous action of the heart and arteries which seeks relief in hæmorrhage.

Consistent with this view, however paradoxical it may seem, we witness in the fearful struggles of an epileptic, the attempt of Nature to cure the disease, or, at least, to mitigate the effects of that morbid condition of the nerves which constitutes the disease; and this opinion is so far corroborated by the fact of the temporary relief experienced by the patient after a fit. It would appear upon reflection, assuming the morbific *cause* to exist in the intervals of repose as well as in the paroxysms (as it must do, not being removed), to be a beneficent provision of Nature, that the morbid irritability of the nerves should, in epilepsy, be discharged by one great explosion, during the unconsciousness of the patient, than escape by frequent, though slighter, shocks, to his constant and conscious distress. We can no more explain how it is that the phenomena of convulsions should display themselves through this or that set of nerves, than we can demonstrate the morbid condition which produces them. It is inexplicable what that change can be, which in a state of health stimulates a youth to the exercise of his muscles in active games, or in disease contorts them with convulsions. We can no more tell the cause of that steady, even flow of nervous energy, which in health produces so many agreeable sensations, than how its changed or interrupted course excites convulsive passions; nor how it happens that the same nervous stimulus should in one state urge us on to noble and heroic deeds, and in another, impel us blindfold to some reckless folly.

Having given, in the above brief sketch, a general idea of the character of these convulsive affections, and of the constitutions of body most susceptible of their attack, the other forms in which they present themselves will be simply enumerated, and the application of the principles already laid down be left to the intelligent reader.

5. Chorea, or St. Vitus' dance, belongs to the same family of diseases as the above. It is simply an exaggerated state of that morbid condition alluded to at the head of these affections, (2.) and there can be no doubt but the nervous twitchings and eccentric movements of a young person said to have "tricks" may, by mismanagement in moral or intellectual culture, be *driven* into an attack of chorea, or some similar disease.

6. That very interesting and remarkable affection, which is seen in catalepsy, also requires extreme care in its management, lest it should lapse into the more serious condition of epilepsy.

7. Somnambulism, likewise, calls for the most careful and judicious treatment, as it is often but a symptom of some greater mischief impending.

In proportion as the improved sciences of physiology and pathology add to our knowledge of the essential elements of health and disease, they cannot fail to excite alarm in every reflective mind at the accidents so likely to accrue to the delicate and mutually dependent tissues of the body, and either to derange their functions in health, or to destroy their structure in disease. If this be found true in the general anatomy of the body, how much more applicable must it be where the minute tissues and intricate fibres of the brain are involved! —an organ so sensitive to morbific influences, that its functions will be impaired, or altogether destroyed, by causes so subtile as not to be detected on post-mortem investigation. But the alarm which a review of these facts would naturally engender is swallowed up in surprise on observing, that the functions of so delicate an organ are not more frequently and more seriously deranged by the turmoil of life, and the incessant demands which commercial and literary competitions make upon our energies. It is indeed true, that the consequences of these excessive mental demands are not always visited upon us in our own persons, but are reserved for more condign punishment in their infliction on our children, who become living and lasting judgments before our eyes, at the same time that they convey lessons of amendment to the present, and of caution to the future generation.

If we more duly reflected how often with a name or fortune we transmit to our children the terrible nervous affections (and sometimes even the fatal seeds of family extinction) which they inherit from the exhaustion of our own energy in the gaining of these vanities, we might be induced to attribute more real happiness to health, virtue, and longevity, than to the pursuit of worldly gain, or to a conformity with the existing errors of our whole social system.

8. Before quitting the subject of convulsive diseases, the present will be the fittest opportunity of making a few remarks on *precocity of mind*, for it appears to depend so much upon a similar condition of the nervous system as to render its classification with those diseases both practical and useful.

The characteristics of this condition of youth are too obvious to require definition. It is almost always associated with a weak physical organization, and a scrofulous tendency, especially pulmonary consumption, proving the correctness of that popular remark, that a child of this precocious habit "is too clever to live." It will be found, too, that these instances of precocity are not indications of *general mental vigour*, but exuberant displays of one or two faculties only, such as memory, imagination, &c., and that the other and superior faculties are more or less weak. Children of ten years of age, or younger, may show a wonderful talent for music, or calculating, or any knowledge in which memory alone is concerned, such as facts of history, citation of poetry, or even languages; but we never find them to present any general mental precocity, in which the higher mental faculties are concerned, such as invention, judgment, conception, &c., they merely afford instances of unusual mental acquisitiveness. It must be concluded, therefore, that in all these cases of precocious intellect, the real state is one of weakness—a state so apt to be overlooked in the admiration we bestow on the brilliancy of one prominent faculty.

Although the following appropriate quotation is made from Dr. Wigan's interesting and ingenious work, it must not be inferred that assent is given to his notions of the duality of mind. Speaking of precocious intellect, he observes—"It is not always possible to keep it in check by the most judicious proceedings, for it is generally caused by a preternaturally rapid growth of the convolutions on the surface of the brain, incompatible with great physical vigour; but unfortunately such premature intellectual powers are so flattering to the vanity of parents, that it is scarcely possible to convince them of the danger of their early cultivation. The child learns with such ease, its acquisitions are made with so little effort, that the fond parent cannot believe in the predictions of the medical friend, and spurs the willing steed to leaps beyond his strength, till the brain fails under the effort. Knowing these things, having witnessed the miserable consequences, I could not read the correspondence between William Pitt and his father without a feeling allied to terror. Never did man go so near to destroy the intellect of his son by over-excitement as the Earl of Chatham; 'Courage, my son', said he, in one of his letters, when the poor lad was complaining of the enormous variety of topics urged upon his attention—'courage, my boy, remember there is only the Cyclopædia to learn!' William Pitt was nearly falling a sacrifice to his father's ambition. Great as were his talents, I do not doubt they would have been much greater had they been more slowly cultivated, and he might then have attained the ordinary term of human life, instead of his brain wearing out his body at so early an age. To see him, as I have done, come into Bellamy's after the excitement of debate in a state of collapse, that with his uncouth countenance gave the air of insanity, swallow a steak without mastication, and drink a bottle of port wine almost at a draught, and be then barely wound up to the level of ordinary impulse—repeat this process twice, or I believe even three times in the course of the night—was a frightful example of over-cultivation of the brain before it had attained its full development. So much had its excitability been exhausted by premature and excessive moral stimuli, that when his ambition was sated, it was incapable of even keeping itself in action without the physical stimulants I have spoken of. Men called the sad exhibition the triumph of mind over matter; I call it the contest of brain and body, where victory is obtained at the sacrifice of life."—*See* p. 305.

In the preceding cases, we have observed a general debility of physical and mental powers to prevail; though frequently disguised to ordinary observers by the apparent vigour they assume under the garb of increased nervous irritability—a morbid condition only to be detected by the experienced physician. It now remains for us to consider that condition marked by—

*2nd. Diminished Irritability of the Nervous System, with general want of Mental and Bodily Vigour.*

1. There is a state of weakness of mind too apparent to escape the notice of any but the indulgent and partial parent. What is usually called imbecility is alluded to—a condition of mind which requires a separate and particular consideration. An imbecile or weak mind is always associated either with impaired health or a low physical organization. A child of weak mind may, to the unprofessional observer, present the appearance of robust health; but to medical men, a weak mind and a sound body are known never to co-exist. By imbecility of mind is to be understood any condition below the standard of ordinary intelligence, and above that of the congenital idiot. The present remarks, however, will be confined to that form of it in which the mind is not so obviously defective, as either to preclude admission to social intercourse, or to debar hopes of future improvement. It is just under such circumstances that fond parents are so ready to ascribe what is really mental imbecility in their children to nothing more than a little slowness, and perhaps idleness fostered by home indulgence; for the effectual cure of which they blindly trust to the stricter discipline and more exciting competition of a school.

In the preceding cases, we have observed a morbid *irritability* of the nerves to prevail with more or less weakness of mind. In imbecility, there is a *defective nervous energy* in addition to the mental weakness—an association which renders it more distinctive in appearance from the former cases than it really is in fact. When imbecility of mind is united to a low nervous condition of body, it is only a more conspicuous state of what exists in the cases described. We are not to suppose, because the subject of imbecility is slow in action, as well as dull in learning, that he is necessarily of a weaker conformation. So far from such being the case, morbid irritability of the nervous system shows even a greater and more dangerous amount of weakness than torpidity.

It is in boys of this slow and dull nervous temperament that we find that occasional *waywardness* of temper which, by mismanagement, so often becomes a sullen obstinacy. Now, if we regarded this perverseness in its proper character, as the symptom of a weak mind, resisting the pernicious impositions which the usages of schools would press upon it, we should be sparing ourselves much needless trouble, and, what is of more consequence, should be sparing the child whom we are educating, much mischief as well as misery. If in these, and indeed in all cases, we followed nature more, and our own preconceived notions less, we should not commit so many fatal errors in the bringing up of youth. If like the intelligent physician, who recognises in every symptom that great president of health, the "vis medicatrix naturæ," the attempt of nature to throw off morbific matter, and who is guided in his treatment by such knowledge; if, like him, the teachers of youth could analyze the nature, the causes, and the motives of that wayward condition in a child, which not only induces aversion to scholastic discipline, but repels, with the utmost obstinacy, every restraint of the kind, they would cease to be empirical, and would be fulfilling their important mission, in a philosophical and more successful manner. A skilful physician does not presume to oppose, but simply to lead symptoms, he only assuages their inordinate activity, guards them from any accident in their course, assists to mature them in convalescence, and co-operates in their retreat on the return of health.

A course precisely similar should be pursued in cases of mental imbecility or disease. The moral disturbances observed in such cases should be regarded as symptoms of a weak mind in a weak body; and our duty is, instead of opposing the designs of nature, therein to follow the example of the physician, and to consider those symptoms as curative means, the "vis medicatrix" which nature sets up; and thus to try if, by taking them as our guide, we shall not be repaid with the same success as attends the treatment of any mere bodily derangement. This is the philosophical and rational view of the subject, though hitherto it has not been taken and acted upon, at least on any extensive scale.

But if we have hitherto greatly erred in our system of education, if we have been endeavouring to bring down nature's inviolable laws to our own artificial rules, as is too apparent to be denied; let us now, at least, confess our past error, and begin to adopt the more enlightened, and at the same time more simple, course which nature points out; let us listen to her sacred voice, pleading with us in the mental listlessness, the bodily indolence, and the moral eccentricities of our children; and let us seek for some other system of education more suited to their state, and more in accordance with nature's own indications than the system now generally in use.

A physician, faithful to his trust, engages in an office of more than common worldly interest; he is the naturalist of his own species, and soaring above all venality, he fulfils the intention of nature's God, whilst acting in unison with nature's laws. The noblest work of the physical world has been committed to his care, and diligent in his calling, he studies the operation of those conservative laws which have been ordained by a wise and benevolent Providence for the preservation of the body in health, and for its restoration in disease. He learns as the result of his studies and researches on this subject, that there is no inconsistency in nature's works, though there is an almost endless variety; he traces in their whole arrangement nothing but harmony and wonderful analogies. This, indeed, is what we might "à priori" conclude would be the case, knowing, as we do, that "nature" and "Providence" are only other terms for the government of a Being who is of perfect order and equity. The contrarieties which we sometimes suspect, are not real, but the fruit of our own ignorance; and the various physical and moral disturbances which afflict us in this world—such as death, pain, insanity—are in as perfect harmony with Divine justice, as with any known natural law.

Nothing could, at first sight, present to human comprehension so great an appearance of contrariety in every essential quality as matter and spirit; and yet how very analogous are they found when more closely viewed and compared? We see the human frame to be furnished with various organs, all to fulfil one great design—the production and continuance of animal life; and we see the mind as liberally adorned with many faculties, all contributing to one great end—the production and maintenance of reason. We, moreover, see the body provided with *double* organs that, in case of accident, the other may discharge the function of the injured one, and the integrity of animal life be always preserved; and we see the mind also endowed with *organs of sense* as well as faculties, whereby a channel may be always open for the supply of mental food, and thus reason, the grand object of the mind, be sustained and nourished.

Such, by way of brief example, is the analogy between matter and spirit, mind and body, as they are found united in man. There are similar analogies likewise between the infirmities or diseases of the body, and the imbecility or derangement of the mind. And what is now contended for is this : that there should therefore be an analogous mode of treatment, whether preventive or curative, in both cases; and that, consequently, education, instead of being one unyielding Procrustean system, should be so varied in its forms as to suit the capacity of each grade of intellect, whereby the weak might not be sacrificed to the strong, nor the strong be encumbered and kept back by the weak. But the fact, alas ! is this—that, although the principles above stated are really deducible from the laws of nature, yet they have never been practically applied, on any large scale, to the education of the young. It may be said with too much truth that "scholarship" is the idol, to which thousands and thousands of children have been, and are still, sacrificed; the pride of parents forbidding them to think that their children can be of weaker intellect than others, and their ambition urging them on to such mental labour and study as far exceed their powers. The result of such a course, as cruel as it is irrational, cannot but be most injurious, both to the individual and to society.

The following very pertinent remarks on this subject are transcribed from the lectures of Dr. John Conolly. Speaking of the connexion of imbecility with incoherent insanity, he observes : "In many cases, there has existed some congenital defect, very little observed in early life, but which becomes declared as youth advances. This is especially the case when girls are the subject of the infirmity. Not being called upon for much intellectual exertion, their great deficiency is for a long time scarcely suspected. They have capacity enough to become skilful in needlework; they go through the routine of school lessons creditably, and sometimes show some skill in drawing, and become accomplished mechanical musicians. But when they arrive at an age in which the affections are expected to be active, and the judgment capable of exercise, they manifest an indifference to their relations, or an indolence, or apathy, or evident want of power to think and act for themselves. They pursue their occupations in an irregular and desultory manner, neglect exercise, acquire odd nervous habits, become negligent in dress and behaviour, are capricious and irritable, and are found to require constant superintendence. In male subjects, the defect is probably earlier suspected; they exhibit a partial cleverness as boys, but with some waywardness, or other peculiarity; and as they grow up, they are remarkable for obstinacy. When they reach adult age, the inequality or disproportion in the mental faculties becomes very perceptible. They can make certain acquisitions in knowledge, even to a considerable extent, and utterly fail in attempts of a different kind; they are perhaps expert calculators, or have a retentive verbal memory, or become proficient in the practical parts of music, but seldom acquire accurate scientific information, and cannot apply continuously to anything. If the circumstances of the individual place him above the necessity of regular exertion, he is only looked upon as eccentric, and he perhaps evinces a shrewd apprehension of the advantages of property, and the value of money. If exposed to misfortune or any agitating circumstances, the mind generally becomes deranged. If placed in various professional situations, such young men leave one pursuit for another, and for a time appearing only unsettled, are at length found to feel no interest in any pursuit. If at college, they will go on making classical acquisitions, but show an utter indifference to engaging in any occupation or profession for which their education was intended to prepare them. Moroseness, irregularity of habits, indolence, and negligence become more and more perceptible; they are easily alienated from their friends, and form unaccountable attachments to strangers. They quit the university in disgust, repudiate divinity, medicine, and law; prove unfit for holding commissions in the army and navy; become suspicious, entertain delusions, and it is seen that they are entirely of unsound mind. Although they have an evident distrust of themselves, and are timid and irresolute, and have often a suspicion of their own morbid state, or a dread of insanity, they are jealous of interference, impatient of being watched or advised, fiercely resist attempts to control them, and sometimes become dangerous to those relations who exert the most anxious care for their protection. There are several cases in which a degree of imbecility of mind is always shown when the bodily strength of young persons is impaired. The brain falls into a condition of debility without insanity. To a certain extent, varieties of cerebral energy constantly accompany the variations of bodily health; and the object of all care, both as regards the sane and insane, and all the gradations between them, is to regulate both body and mind in such a way as to ensure to each individual the extent of cerebral power of which his organization is capable. Any debilitating cause may bring on a temporary or permanent imbecility in young persons of delicate constitution and feeble organization; neglect of exercise, or over-exertion, or too low a diet. When the health improves, in consequence of the removal of such causes, the mind becomes stronger, and the patient talks, writes, and acts rationally."

Having now attempted to give a popular and familiar description of those conditions of the nervous system, which render the modern plan of education so detrimental to the happiness and virtue of many young persons, as well as so defective in its object of instruction, let us proceed to examine in what way it is detrimental, and for what reason it is defective.

First, in what way is the modern system of education so detrimental? The answer to this question must be first prefaced by a few remarks on the nature of mind. For the great end and object of education is to produce a healthy and well-informed mind, which shall be a source both of pleasure to the individual, and of advantage to society at large. It must, therefore, be a point of primary importance, that they who pretend to educate should have some correct knowledge of what the mind really is, and how it stands related to, and dependent upon, both the soul and the body in man.

In every one that is born into the world, there is this unseen and incomprehensible union of spirit and matter, soul and body; and this union continues until the death of the body dissolves the bond. Now, after all that has been written on the nature of mind, the whole mystery seems reducible to this simple interpretation, that "mind is the manifestation of the soul through the medium of the body." There must be a human soul operating in a human body to constitute mind. Its phenomena continue only during the lifetime of a man; at death they cease—the body then returning to its kindred earth, and the spirit to God who gave it. Divine revelation has not declared much to us respecting the state of the soul in its *separate* existence, beyond the fact of its spiritual intelligence, and its final subjection to reward or punishment; but experience reveals to us much of its nature when united to matter in the attributes of mind. Mind, then, is the effect of that mysterious union. It can take cognizance of external objects only *through* the body, (in the organs of sense;) it can display its internal sentiments only *through* the body, (in speech, writing, acts;) and it can reflect within itself, in its own secret chambers, (in thought, conception, &c.,) but still *in* the body; hence its restrictions. We see, therefore, that the mind is not identical with the soul; nor is it a mere bodily function, as the materialists affirm; but an independent quality—the offspring of the union of soul and body, the special seat of that union being in the brain. We see, too, that it partakes of the mixed nature of its authors in its spiritual conceptions, which come directly from the soul, and in its more physical perceptions as well as in its animal sensations, which are derived from the body; that it continues only whilst the union lasts, and ceases at their separation, to return when they shall be reunited in perfection to partake of their refinement.

Limiting the word mind, then, to express our meaning of those phenomena which are the consequences of the union of spirit with matter, soul and body—phenomena which partake in a mysterious manner of the nature of both these component parts in man, we can establish some certain data whereon to explain the reciprocal derangements which disease in the one will establish in the other; the mind equally participating in the diseases which may affect either of its parents—the soul or the body.

But how can the soul be diseased? Every departure from virtue is a disease of the soul which will affect the mind, and through it, more or less, the body. Again, there are some lesions of the body which, according to their extent, will affect the mind in one or all its faculties—from a simple derangement of some of its most physical manifestations, as seen in eccentricity, monomania, &c., up to its highest intellectual and moral faculties, as in mania, melancholia, and moral insanity; and we have, accordingly, some fair guide in determining the responsibility of insane people, as their unsoundness of mind shall spring from the former or latter cause—as their derangement occurs from a primary disease of volition, which they have themselves occasioned by vicious indulgences, or a primary disease of the body, over which they can have little or no control. It must, of course, be always a difficult matter for fallible man to demonstrate the applicability of these facts in individual cases; and the real truth must, therefore, in many instances be concealed from us till that day when all the world shall be assembled before the judgment-seat of God.

It is now pretty generally allowed that the brain is that portion of matter in which the mind resides, and the nerves those organs through which sensations and volition are conveyed to and from it. Assuming this to be the case, we may not inappropriately regard the brain, when in perfect health, as a clear glass through which all the nobler qualities of the mind show themselves, and by which it perceives, apprehends, and reflects upon all kinds of objects. Let us extend the simile. If we take a piece of glass and expose it to the flame of a lamp till it become blackened, or disfigure its surface in any way, we find the light of the sun to be diminished, or its rays distorted, according to the amount of opacity or disfigurement produced.

It may be presumed that disease exercises an analogous effect upon the brain, whereby the mind displays itself in a disordered manner, even to hallucination and fatuity. Entertaining these views, with what a reverential awe must the devout philosopher be impressed when he understands how an immortal spirit tied, whilst on earth, to a corruptible body, becomes subjected in its character as mind to the physical laws of matter!—with how much anxiety must he regard that most tender and sensitive organ, the brain, composed of such delicate fibres and minute vessels, when he learns that it is the seat of human reason! He knows how slight an accident will destroy its structure, and render it unfit for its purposes in our economy; and observing how jealously nature has inclosed it in a strong bony case, and protected it beyond any other organ, concludes that, as it is the most delicate and perfectly organized work in nature, it demands our utmost exertions to preserve it from injury in every stage of its development.

Interesting as it would be to prosecute these important inquiries, we must enter upon them no further than as they bear upon the subject of education.

Such, then, are our views of the union which subsists between spirit and matter, soul and body, and of the results of this union, as seen in the various operations of the mind; and harmonizing as these views do, and as all scientific deductions ought to do, with Divine revelation and human experience, the applicability of them as a guide to some sound and rational system of education demands our confidence and excites our hopes.

It will be readily allowed by all that the first and great object of education should be to establish a healthy mind in a healthy body, to assist nature in the development of every mental faculty and corporeal power. This being kept always in view as the first and great object, the next will be to supply, by every suitable means of instruction, the largest amount of the best and most useful knowledge which each particular mind can with safety receive.

These should certainly be the principles acted upon, and the objects aimed at in the education of youth. But if any departure from these first principles in education be accompanied with correspondent mischief to youths of ordinary capacity, how much more positively injurious, and even fatal, must it become to those whose mental and bodily temperament agrees with any of the conditions already mentioned.

From the intimate relationship existing between the mind and the body, we cannot be surprised at the exquisite sympathies which spring up between them, causing those mutual actions and reactions which produce so many inconstant sentiments, and which will so often depend on the healthy condition of the individual whether they be pleasant or painful sentiments, cheerful or apathetic, that we may almost proportion the amount of disease to the intensity of these moral disturbances. How defective must that system of education be which is not founded on these known laws of nature, and which is for the most part conducted by teachers, who, though they may have read many books, yet have never made man their study. There need be no hesitation in asserting that the modern system of education is conducted upon principles entirely empirical—that even common sense views of it are sacrificed to absurd custom and bigoted prejudice. It is a system, indeed, which proves in general as preventive to the acquisition of real knowledge in the pupil, as it is detrimental to the morality and happiness of the future man. That it sets up its own uniform but coercive standard to which all minds must succumb, in the attempt to attain which the weak break down, and the strong having attained it, are too much exhausted to go beyond. And why is this? Simply because we try to adapt the faculties to knowledge instead of knowledge to the faculties.

Now, it so happens that the sphere of life in which the author of these remarks has been called to move and act, has been among the victims of this false and injurious system, and that for many years scarcely a day has passed but he has been brought into constant domestic intercourse with many of those pitiable objects, among the upper classes of society, who having in childhood only one talent, were robbed of it at school, from which time it has lain buried in ignorance, useless to themselves, and lost to society. He is relating, therefore, matter of fact and experience, and he knows that there are others in this kingdom, engaged as himself, who can authenticate the truth of what he has affirmed.

It does not require much acquaintance with the laws which influence the accession and progress of disease in the human frame to comprehend the general disturbance which the dry, uninteresting, routine of scholastic pursuits must set up in constitutions so prone to irritability as those already described. Our present system of teaching being an entirely coercive one—being founded on the principle of diverting all the faculties of the mind into one common stream of knowledge, admitting of no natural tendencies, allowing no deviation from established rule, exacting from the weak more than they can contribute, and restraining within these arbitrary limits the more vigorous and enterprising, is alike injurious to boys of excessive or defective talents.

There are as many streams of knowledge as there are mental faculties; and to dry up the natural springs of the mind, in the attempt to bring them into one common channel, is an act as pitiless in its means as it is frustrative of its end. What natural tastes, or mental inclinations, are sought for in the youthful mind at school? or being found, are cherished and duly cultivated, and made the medium of conveying knowledge to the mind? Experience tells us that not only is such a proceeding never thought of, but one so totally opposed to the object is adopted, that it would seem to be the end and aim of schools to thwart nature at every turn, and to make her succumb with the pupil to the artificial scheme which they collectively agree to support. We find that, whenever an ill-appropriated system of teaching disturbs the harmony which should exist between the operations of the mind and the various functions of the body, disease is in some form immediately established, which, displaying its effects upon the weakest structure, will accordingly show itself in some form of irritability in subjects of a weak, nervous temperament. We have then a disease established. Now, one of the universal laws of disease is, that it will continue and increase so long as the cause remains. We may, therefore, easily surmise how the same cause, being persisted in, which excites this irritability, though the ill effects may at first be trivial, yet may be augmented into one that will ultimately produce the most alarming results.

The equilibrium is first only disturbed, then excited, then weakened, then exhausted, and finally destroyed; and we may trace in this gradual decline of power the concurrent freaks and irregularities seen in the inattention, the idleness, the apathy, the perverseness, and the various immoralities of the weak-minded schoolboy, and which spring up as much from impaired general health as from defective intellectual and moral energy.

If the views now propounded have any claims to accuracy, it behoves us to remember that whenever indolence, idleness, listlessness, obstinacy, perverseness, or any moral delinquency, as excessive cowardice, excessive petulance, lying, stealing, or any particular depravity exist, before punishment be had recourse to, the cause should be thoroughly investigated by a competent and experienced person; that if it cannot be attributed distinctly to a *wilfulness* springing from *acquired* corruption, (the natural talents being of a superior order, and the bodily health good,) then the fault lies in the education and not in the pupil; that punishment would be an aggravation of the evil, that a change of system can be the only efficient corrective; and that just as the severest corporeal punishment would be the proper correction of *folly* and *wickedness* in the one instance, it would only harden the heart and confirm the *disease* in the other.

In the absence of good abilities and good health, all these moral vagaries should be regarded as the consequences of weak intellect and deranged nervous energy, aggravated by mismanagement—as symptoms of a real disease requiring as much skill and experience in their treatment and cure as fever or insanity, or any other malady.

Assuming, then, that almost every departure from an ordinary amount of mental and bodily strength is a condition of more or less disease, it would be just as consistent to insist upon a patient to eat and drink, and walk out, and transact his usual business in fever, or any other serious bodily disorder, as to apply the same system of education, and to expect the same results, in youths of defective powers as from those of robust health and ordinary talents. We will not, indeed, dispute the soundness of the principles on which the education of those having ordinary or superior abilities is established, if it be admitted that every condition of mind short of these being dependent on disease, should justify a departure from that system which is avowedly adapted and intended only for a healthy state. We will not stop to inquire how far the whole system is founded in error, if it be only allowed on the evidence of the foregoing remarks, that it is totally incapable of fulfilling its great designs in the subjects of these nervous derangements. Nay, we may go so far as to grant that, till the principles and habits of modern society be themselves reformed—till the competitions, the avarice, and the intense selfishness of the age yield to more primitive and temperate ideas, education, as now conducted, is, to a certain extent, necessary to keep the human mind up to the high pressure point of this impetuous epoch of the world.

Can anything be conceived more irksome to a boy of slow apprehension, than the very first lesson he is taught at school—the definition of the parts of speech—the dry grammatical construction of language? Can anything be conceived more *exasperating*, if, in addition to his natural dulness, he possess a highly nervous susceptibility, and perhaps some vivacious sentiments, than the daily repetition of a task he can neither understand, nor perhaps has even verbal memory enough to repeat to the satisfaction of his teacher? Can punishment elicit from one of such a conformation an amount of intelligence which he really does not possess! And yet it is expected, because it happens to be the custom of the school, and in the usual order of teachers. In all such cases, there can be no doubt in the mind of any impartial person, where the error lies—it is in the system, and not in the pupil; it is in the scholastic pride, which being ignorant of, or disregarding, the laws of nature, would supercede them by its own artificial rules—it is in the folly, or something worse than folly, of sacrificing the moral interests of the individual to a vain attempt at forcing him to an intellectual position which Nature has denied him.

Here, then, is the conclusion to which we are brought; and as we believe, by incontrovertible facts, which are the best of all arguments; that in a great number of children there are certain morbid susceptibilities, which render the present general system of education not only futile as a means of imparting knowledge, but absolutely injurious both to the minds and morals of its victims. The question, therefore, arises, what other method can be adopted to supply those great desiderata of our present life, a healthy body, sound morals, and a well-informed mind, which may be regarded as the three great sources both of individual and of social happiness. To effect these important ends, not only must schools, but every other substitute partaking of their arrangement and discipline, be avoided. The principles of education must be altogether different. Both the *amount* of knowledge to be imparted, and the *means* of imparting it, instead of being one and the same for all, must be varied according to the variety of cases brought under instruction, the mental food being in all cases suited to the powers of mental digestion. In fact, we must establish new institutions, wherein all cases of defective mental powers shall be regarded as *patients* as well as pupils. A judicious medical treatment must prepare the way, and go hand in hand with the schoolmaster—an experienced medical man, who has made the human mind, in its healthy and diseased states, his study, must watch these cases, and according to the degree of mental and bodily health in the pupil, must apportion the quantity and quality of instruction to be given; must gain by little and little upon the weak mind; feeding it sometimes with only crumbs of knowledge; and thus discreetly proceed, day by day, till his toil and care shall be repaid with the satisfying consciousness of having saved a fellow-creature from all the evils attendant on ignorance and vice, and of having been the instrument of adding a virtuous member to society.

We are informed by a higher than any human authority, that "the fear of God is the beginning of wisdom;" and this is indeed admitted as a general truth, but, alas! is practically neglected in most schools. There can be no doubt, however, that unless it be made the groundwork of early education in these peculiar cases of which we speak, then the most careful watching, the most gradual introduction of knowledge (even through proper channels), will certainly fail and disappoint our hopes.

Next, therefore, to the establishment of good bodily health by a judicious medical treatment, the production of a healthy moral tone must be sought for through the medium of religion, by instilling into the mind those sound principles and motives which Christianity furnishes, and by inculcating those pure and holy precepts which it lays down for the regulation of daily life. It is remarkable, too, and evidences the wisdom and goodness of God, that there should be such a connexion and reaction between health and morals—good bodily health, on the one hand, conducing so much to promote morality; and good moral habits, on the other hand, tending so much to promote bodily health. Where both these results can be obtained—viz., good health of body, and a healthy moral tone, it presents the best possible basis on which to carry intellectual cultivation. It may, indeed, and often does, happen, that these three processes of improvement (physical, moral, and intellectual) may go on together under judicious management, and the one assist the other; but it should always be borne in mind, that the above is the natural order, and that whenever it is reversed, by seeking intellectual advancement at the expense of health and morals, it must be to the certain injury both of the individual himself, and of society at large. For it may be laid down as a fixed law in political science, that society is never so fundamentally benefited by excessive intellectual advancement, as by the less showy but more wholesome spread of virtue. It is the greater amount of virtue which renders one country more prosperous and powerful than another. Had the French, German, or Italian people been as conspicuous among the nations of the world for private virtues, as they have ever been for intellectual genius, the wave of Revolution, which has shaken or destroyed their institutions, would have passed over them as harmlessly as it has broken upon our own more happy shores. The cultivation of the moral faculties, therefore, must form a leading feature in our system for the development of weak intellects, and the daily practical exercise of those faculties, reacting on the general health, will prepare the brain and nervous system to bear with impunity, and even advantage, the importation of knowledge.

Of all the moral faculties, *chastity* is the most invigorating to the growing youth. It is a fountain of inward nourishment, which circulating through all his thoughts, words, and actions, adds strength to body, mind, and soul. If its source be checked or tainted in the slightest degree, there will be a correspondent disturbance in the physical and moral growth of the individual. Chastity is the key-stone of the moral arch; and the whole fabric will stand or fall, according to the integrity of this important particular. It is the parent of good taste, of refined mind, and correct manners. It is the great source of individual happiness and health, as well as indispensable to domestic peace and social order—and without it, how can there be admission into that celestial city where all is pure and holy? Seeing, then, the influence which this blessed principle exercises over the physical and moral welfare of mankind, how lamentable that it should so often be left to chance, whether it shall grow up and bear its own lovely fruit, or wither and die, and give place to some of the worst passions of our fallen nature. But at the same time, it may well afford great encouragement to the educators of youth to know, that there is such a faculty for their cultivation in those committed to their charge; and that the culture of it, if successful, will be repaid with such wholesome fruits.

But if the cultivation of the moral virtues, of which chastity is the chief, be essential as an element of early education, much more the cultivation of religious principles, the chief of which, and the ground-work of all the rest, is *faith*. The condition of mind which a high moral tone and religious principle induce in youth, is just that condition best calculated to promote the growth of a vigorous and healthy nervous system. The bodily frames of growing young people require the utmost immunity from the disturbing agencies of a moral kind, if, at least, they are to be made the temples of virtuous sentiments. It is as much impossible for the youthful frame to grow up into all the graces of manly excellence in a moral atmosphere agitated by debasing passions, as for the sapling to become a gigantic oak on the blustering sea-coast. We accordingly find that just as these elements of spiritual life are wanting, we for the most part observe that the vital tone of the body will also decline, till at length spirit and matter will find their low estate in the fellowship of painful bodily diseases and agony of mind.

That too little attention has been paid to the culture and proper training of Faith in childhood (always so prominent a feature at that age) we have only to look around on the existing and increasing amount of infidelity in the world, and to observe its influence and effects on those who profess it, in their wicked course of living, and unhappy deaths.

Religion cannot be disregarded by any one with impunity; and though a few men of hardier temperaments, and of more audacious minds than others, may indeed do so with apparent worldly prosperity, and that, too, in conjunction with an outward form of respectability, its supporting influence cannot be rejected by those of weaker conformation, without disgrace to themselves, and ruin to their prospects.

It becomes, therefore, of paramount importance, on the first dawn of reason in the imbecile, to make available this youthful characteristic to the purposes of establishing religious principles, which cannot fail but to invigorate as they are imbibed, and to prepare the way for confirming that supremacy of the Will which, in the future activity of manhood, shall dictate unconditional terms to temptation. It is in the early age of childhood, when the citadel of the heart is as yet ungarrisoned, and an implicit faith opens the door alike to friend or foe, that we must take possession of, and hold it, till the armoury of life's warfare be furnished with sound principles, and we be relieved from our watch-guard by the spirit from on high. It is now that all the trials, the dangers, the misery, and the ruin, which haunt the unguarded path of such children are to be averted. It is now, at this early age, for parents to determine whether their children, susceptible (through constitutional infirmity) of such deep and such lasting impressions, shall be exposed to those risks which the more vigorous often fail to escape, or be imbued with those precepts which give grace to the simple, and confound the wisdom of the world. It is only in the early cultivation of a sound religious faith that a prevention and cure is to be found for those blind impulses of youth—for that reckless indifference to life, health, and virtue, which they exhibit in their course through the world, and which hurry them on in their infatuated career to infamy, madness, or death.

These moral and religious influences, acting through the agency of the mind, operate beneficially upon the body; for it is by inducing a regularity and correctness of thought, as well as by establishing a proper control of the will over the passions, that they ultimately react upon the animal tissues, and improve the tone of the whole nervous system.

But besides these, there is another influence to be enlisted in our service, which though more physical in its action, is scarcely less beneficial in its effects—viz., Habit.

Perhaps nothing requires so much care and judgment in the management of the imbecile as this very important instrument, especially when applied to moral or intellectual purposes. For such children are so apt to resist (through natural instinct) all kind of restraint, whilst tutors are so partial to the use of even *con*straint with their pupils, that the danger generally lies in our attempts to push Habit too far than otherwise.

But the same objection does not obtain in its application to the bodily regimen; for in the regularity of diet, sleep, exercise, amusement, &c., the utmost precision should be practised—a compliance with which is the more readily yielded from the personal comfort experienced in adopting it, with this additional advantage, that we gain upon amicable terms the important point aimed at—viz., the introduction of Habit by the practice of a regularity that is agreeable to them.

How much mischief is done in early childhood by our neglect of a judicious discipline, and by the loose manner in which we enforce filial obedience! The remark is in every one's mouth, and its truth must be admitted by the most thoughtless observer; and yet the consequences of these early defects, although vividly enough portrayed to the parent in family discord, and to the statesman in the revolution of a country, nevertheless excite amazement when they really do come to pass. But these consequences can be traced to their real source by those who have learnt that no moral delinquency can happen without imprinting on the structures to which the soul is allied some mark of injury, which by a too frequent repetition of the cause of injury may become so irremediably impaired, that were the mind even disposed to do right, it could find no healthy medium through which to exercise itself.

Mysterious and awful consideration! To know, as the study of disease seems to point out, that there are certain limitations to which the operations of mind and matter are confined, after trespassing upon which there is no retreat; that the bodily organization may be so impaired by certain physical disturbances, or by long repeated moral delinquencies, as to preclude the manifestation of sound reason, and of healthy will, is a humiliating but instructive circumstance.

With these facts before us, we are bound as peaceful citizens, and by the selfish motives of policy alone, to secure quiet in our own homes, and order in the state, by subjecting our children at an early age to the influence of parental authority. But seeing, as we do, the terrible retribution in the flesh, which is visited upon the child through the grave moral offences which spring from a dereliction of this duty in the parent, we are bound by every Christian obligation to avert such consequences by the remedy we possess.

The excessive luxury of the age, in which science even descends from her eminence to pander to its wants, and in which ease or a freedom from responsibilities is the common object of all, will account in some measure for the reprehensible indulgence we not only show our offspring, but even extend to criminals. It is easier and less painful to our luxurious sympathies to indulge the child than exact obedience—to pardon the criminal than respect the law. And shrewd as the age may be in the relative value of profit and loss, it is short-sighted enough not to perceive that it only compounds a present sentimentality for future disaster.

If, therefore, parents are remiss in the discharge of their first and most important duty, and if such evil consequences do ensue from their neglect, it becomes incumbent on them to consider whether those children, at least, who are not gifted as others to combat these accumulated dangers, shall be wantonly exposed to them, or be transferred to the care of such as can discharge the obligations they have themselves repudiated.

It would far exceed the intended limits of these remarks if a detailed account of the medical, moral, and mental treatment that should guide us in the management of these cases, were entered into; nor would it be possible to give more than a general outline of a method which must necessarily vary with the varying circumstances of each case. Indeed, it would be to a certain extent imitating the system so much condemned, if we were to lay down precise rules, or enforce one uniform discipline; for the management of each particular case must be left entirely to the experience and good sense of the physician, who must seize the opportunities of education as they spring up under his own judicious treatment.

It is obvious that they, whose profession and daily business it is to observe and treat those organs and faculties when diseased, must best know and most duly weigh their capabilities and power of endurance when weak; we say, then, that to such men, combining the physician with the instructor, should the work be committed—to such men should be given the power of ordering the quantity, the quality, the method, the season, and, indeed, every circumstance connected with the important duty of educating a weak-minded yet immortal being. Such men as these alone can fully appreciate the deplorable effects of over-education in weak subjects, and they alone are competent to carry into successful practice the hygienic system now proposed. Such men, conversant with these facts, would at once join in the recommendation of making green-fields the chief school-room, and nature the chief lesson-book for such weak minds; and would study to make the way to knowledge easy and pleasant by means of familiar colloquy, kind sympathy, and good example. Such men, conversant with the various faculties of the mind, would best know how to discriminate between those which may be cultivated with impunity, and made the instrument of strengthening and developing the others. Such men alone can know when to desist and when to persist in their attempts to instruct.

A good foundation of bodily and moral health being established by proper medical treatment and domestic discipline, we may now begin to cultivate the intellectual faculties with general advantage. In some cases of nervous irritability, the five senses may become valuable agents in communicating with the mind; indeed, in extreme cases, they must continue, for a long time, to be the *sole* agents of instruction.

The organs of sense are the tutors of Nature's own providing, and invaluable do they become, not only as coadjutors, but as guides to us in the education of the imbecile. No pedagogues are they! With a wonderfully intuitive precision, they adapt their instruction just to the capacity of their pupil, and, like morning stars, show us where the day-spring of intelligence is about to break. In their curriculum, no pedantic schemes will be found—no unintelligible theories—no dry routine, but the great truths which creation unfolds to the mind.

These, then, should be our great auxiliaries. It is through these easy channels that we are to let knowledge drift into the weak mind, and to instruct, from simple proximate principles, up to more complicated inferences. We must with them begin with the bare impression of an object on the organ of sense, then proceed to induce a right perception of it, till we gradually arrive at the highest mental quality —viz., the conception of original ideas.

We see what important tutors the senses may become, and how beautifully one power may be made to supply the place of another, in the instance of blind people, who can be taught to read, and to know the properties of matter (even of colour) by the cultivation of one of the lowest senses—that of touch. In mental imbecility, there will generally be found some faculty weaker than the rest, as attention or memory—a defect which may often be remedied much in the same manner by judiciously substituting some stronger faculty to perform the duties of the weaker, and which is, indeed, to a certain extent, carried out in the foolish attempt to form an artificial memory.

It is by thus dividing the burden equally among the faculties, imposing upon none more than it can safely and agreeably bear, and by keeping some even in a state of repose, that so much may be accomplished in educating the weak-minded, and bringing them gradually to the possession of that kind of knowledge which, though it may not make them eminent in literature, will at least make them competent for the useful and respectable business of life. And, moreover, we have always this consolation in store, that however deficient such may be in mental acquirements, there can be no excuse nor reason why they should not become even instructors to their more talented brethren in their exemplary moral conduct.

It has been previously suggested, that an improved system of education would be best obtained by discovering the natural bias of the mind, and adjusting the quantity and quality of instruction to the scale of the understanding; but in this selection, a judicious discrimination cannot be too much exercised in distinguishing the depraved taste which the indolence common to weakness is so apt to engender, from the prominent mental or moral faculty which forms the character of the individual. We must be careful not to confound *acquired* bad taste with *natural* inclination, and thus cultivate what it should be our duty to suppress.

As curiosity, or the spirit of inquiring, is the first evidence of mind in infancy, and the last of all to succumb to the assaults of age or disease, so is it always to be found wherever any trace of mind exists.

This inherent principle, ever busy, will, when left to self-tuition, range from lofty speculations to grovelling views, according to the accident of circumstances or ability; and the mental listlessness so frequently seen associated with imbecility is, perhaps, (as previously suggested,) more frequently the effect of an aversion to our artificial rules than of a defective nature. How often, for instance, do we find the listless pupil, however idle in the school-room, only too busy and apt a scholar out of it, in plotting mischief or in reading vicious novels? in all of which he is as much seeking relief from the tedium of school drudgery as yielding to the influence of a spirit of inquiry, and of a desire for knowledge.

The effects upon the health, both of body and mind, which arise from the morbid imagination, caused by reading immoral works of fiction, are most disastrous.

The Arab, in his native desert, pursuing his dreary path, will pass whole days in abstinence, allaying the pangs of hunger with opium only, and, exhausted at his journey's end, sickens at food. The poisonous drug betrays him; and loathing the staff of vital aliment, he ever after yields to a fascination that leads him on to misery. Even so is it with the case now before us. The natural craving of the mind is for knowledge; and when the mind is healthy and vigorous, it delights in the wholesome food of facts; but when weak, in fiction.

We can, however, convert this failing in the weak-minded to their own advantage; for, seeing the ease with which they acquire injurious and fictitious knowledge by the aid of imagination, we can direct them to a study which is more romantic in its facts than any fiction —the philosophy of nature—and in the study of which the pleasure of imagination can be secured, not only without any injury, but with positive advantage to the moral and intellectual faculties. We may safely affirm, that of all the various kinds of mental food most suited to a weak mental digestion, the study of the natural sciences is by far the best. We should leave the pursuit of classical literature, and the more abstruse subjects of moral philosophy, to be engaged in by minds more powerful and more qualified by natural taste to grapple with them.

The rudiments of botany, geology, and chemistry—a general knowledge of the structure and properties of the animal kingdom, of the sidereal system, and of geography—in fact, of all the physical sciences, will constitute the best means of engaging the attention and developing the various faculties of the imbecile. The perusal of history and of select biography will also be found of much service; tending to store them with useful and practical knowledge, and also to form the future character of the man; while the elements of arithmetical proportions and mathematics will engender the precision so indispensable to business and correctness of thought. Indeed, whatever kind of knowledge requires, in the first instance, the use of the senses, in order to its being perceived before it can be comprehended, will be found in all these cases to be the only practical method of fulfilling the great designs of education.

The foregoing observations must, of course, be regarded simply as the introduction of a subject as yet scarcely opened, but presenting a wide field for useful and interesting investigation. Brief, however, as they are, they knock at the door of every family; and if parents and teachers would but canvas the above facts—if they would but enlarge upon the subject now introduced to them, according to their respective opportunities of observation, and thus enter cordially into it as one of the great social questions of the day, some good practical results might then be expected.

But if parents neglect to do this—if those to whom the education of our youth has been intrusted are themselves indifferent in the matter; if their present system is contrary to the dictates of sound reason, and opposed to the laws of nature, and comes short, as it surely does, of the designs of Christianity—such neglect on their part should not deter the medical man from impeaching both the system and its defenders when the penalties fall upon those whose mental and bodily infirmities call forth his peculiar sympathies. Regardless of sectarian pique, it is his duty, as the guardian of public health, to raise his voice against those social evils which impinge upon his stewardship, and to crush in the germ those pernicious influences which fructuate in incurable maladies.

However deplorable it may be, it is nevertheless true, that the modern system of education, instead of being the nursing mother of all that should be esteemed as really great and virtuous among men, is such as to call forth the censures, not only of the moralist, but also of the physician. The abettors of our present system may, indeed, try, and with apparent plausibility, to hold it excused from having caused the various delinquencies of youth, and to shelter themselves under the plea of "free agency" and "individual responsibility," but they cannot establish such flimsy excuses in the cases of those who are the particular subjects of these remarks.

The moralist can only lament effects, which are obvious to all, and attribute them to causes which, though they may be real, can be easily and abruptly denied by any captious disputant; but the physician can deal with the subject in a more palpable way, and bring the naked truth to view, by pointing his finger to the indelible accusation which erroneous education writes upon the living body in the characters of disease.

It is here that education comes within the province of the physician. It is in these particular cases that he has to protest against a system under which the individual languishes and our race degenerates. It is when he observes that the system, in compliance with the folly of an age in which velocity is the criterion of perfection, goads the mind during the tenderest years of life to an activity beyond its strength, at the risk of functional life and moral excellence, that he feels bound to plead for an exemption on the part of the imbecile from that false system, and to use all his influence to save them from those terrible conflicts between mind and matter, for which their frailty so obviously unfits them, and out of which they can only come broken and ruined.

